# AGING COUPLES’ SATISFYING RELATIONSHIPS AND WE-TALK PROMOTE CARDIOVASCULAR HEALTH DURING CONFLICT

**DOI:** 10.1093/geroni/igac059.1383

**Published:** 2022-12-20

**Authors:** M Rosie Shrout, Stephanie Wilson, Megan E Renna, Annelise A Madison, Janice K Kiecolt-Glaser

**Affiliations:** Purdue University, Lafayette, Indiana, United States; Southern Methodist University, DALLAS, Texas, United States; University of Southern Mississippi, Hattiesburg, Mississippi, United States; Ohio State University, Columbus, Ohio, United States; Ohio State University, Columbus, Ohio, United States

## Abstract

Marital conflict poses health risks that intensify as couples grow older. Dyadic stress theories suggest spouses’ marital satisfaction and communication patterns alter cardiovascular function, a key pathway from troubled relationships to poor health. Despite these risks, older spouses are more likely to have a strong couple identity where they think and talk in relational terms. This communication pattern, termed we-talk, is shown when spouses use words like “we” rather than “you” or “me,” reflecting that they are thinking about resolving conflict as a couple rather than as two separate individuals. We examined how both spouses’ relationship satisfaction and we-talk reduced conflict’s cardiovascular toll, and if the health benefits were greatest when both spouses were satisfied and used we-talk. Married couples (n=107) ages 40-87 engaged in a 20-minute conflict discussion while wearing heart rate monitors to assess heart rate variability (HRV), a measure of cardiac flexibility. Couples’ conflicts were transcribed to measure we-talk, or the proportion of first-person plural pronouns, such as we, us, and our. Results showed a person’s HRV was higher and thus healthier when both spouses were satisfied and their partner used we-talk more often. In contrast, HRV was lower and less healthy when neither or only one spouse was satisfied and their partner used we-talk less often. Thus, a couple’s mutually satisfying relationship along with a partner’s we-talk provided a health advantage during conflict. Talking in relational terms may help reduce conflict’s biological toll in aging couples, particularly when their relationships are satisfying.

